# Importance of non-pharmaceutical interventions in the COVID-19 vaccination era: A case study of the Seychelles

**DOI:** 10.7189/jogh.11.03104

**Published:** 2021-09-18

**Authors:** Thomas N Vilches, Pratha Sah, Elaheh Abdollahi, Seyed M Moghadas, Alison P Galvani

**Affiliations:** 1 Center for Infectious Disease Modeling and Analysis (CIDMA), Yale School of Public Health, New Haven, Connecticut, USA; 2 Agent-Based Modelling Laboratory, York University, Toronto, Ontario, Canada

The Republic of Seychelles is an archipelago of 115 islands in the Indian Ocean with a population of approximately 98 000. As of June 28, 2021, the Seychelles was one of only a dozen countries that had succeeded in fully vaccinating more than half of their population against COVID-19 [1]. The Seychelles began its vaccination campaign on January 13, 2021, with two-dose Sinopharm and AstraZeneca vaccines. Both vaccines reportedly have at least 78% efficacy against symptomatic disease 14 or more days after the second dose [2,3]. With various non-pharmaceutical interventions (NPIs) in place (Figure S1 in the **Online Supplementary Document**) and mounting vaccination coverage, the Seychelles suppressed its incidence to an average of 42 daily cases from January 1 to April 15, 2021. By May 5, over 61% of the population was fully vaccinated. Despite the high vaccination coverage, the country experienced a surge of COVID-19 infections soon after most NPIs were lifted in mid-April, reporting the world’s highest number of daily cases per capita and raising concerns about the efficacy of the vaccines [4]. To understand the determinants of the recent surge, and the impact of the interplay between vaccination and NPIs, we used a previously established data-driven dynamic model [5] and calibrated it to reported cases and vaccination rollout in the Seychelles (Appendix S1 in the **Online Supplementary Document**).

We used a Bayesian non-parametric approach to back-calculate the times series of infections based on the daily reported cases of COVID-19 in the Seychelles from October 21, 2020, to May 24, 2021 [6]. We then fitted an agent-based model of COVID-19 transmission (**Online Supplementary Document**) to incidence derived from back-calculation from October 21, 2021, to January 6, 2021. In this period, there were no school or workplace closures, and non-pharmaceutical interventions for stay-at-home, and cancelation of gatherings remained mainly at the ‘Recommended’ level. Border restrictions included a ban on high-risk regions for international travelers (Figure S1 in the **Online Supplementary Document**). We determined disease transmissibility by fitting the model to incidence data during this period while accounting for mask-wearing and recommended measures to reduce contact patterns to 80% of the pre-pandemic behaviour. The model simulated scenarios of COVID-19 incidence without vaccination, and when vaccination was implemented on January 13, 2021 (Appendix S1 of the **Online Supplementary Document**).

We found that adoption of NPIs had been instrumental in reducing COVID-19 incidence from January to April 2021 (**Figure 1**). Without NPIs, the model projected that the country would have experienced a significant early outbreak despite the rapidity of vaccination rollout. The projected surge would have likely led to the infection of over 25% of the population and caused as many as 747 (95% Credible Interval [CrI]: 657 – 832) daily cases at the apex (**Figure 1**, and Figure S2 in the **Online Supplementary Document**).

**Figure 1:**
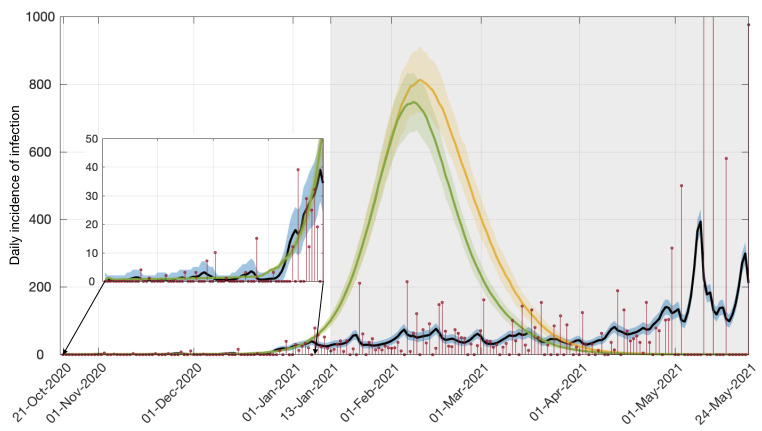
Daily incidence of reported cases (red) and inferred infections (black) with the 95% credible interval shown by the blue shaded area. Orange and green curves represent simulated mean daily incidence without NPIs in the absence and presence of vaccination, respectively. Grey area represents timelines of COVID-19 vaccination in the Seychelles from January 13, 2021.

**Figure:**
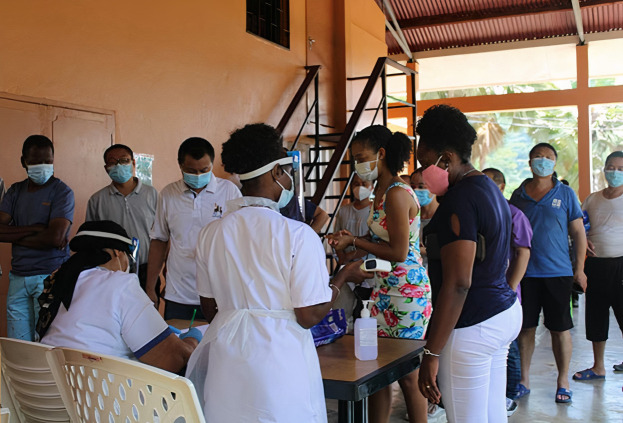
Photo: From the collection of Rassin Vannier, Seychelles News Agency, Licenced under Creative Commons 4.0.

From mid-April to May 4, 2021, school and workplace closures were lifted. The only NPI remaining was the recommendation to limit the size of social gatherings (Figure S1 in the **Online Supplementary Document**). The ensuing precipitous increase in COVID-19 infections highlights the importance of continued adherence to NPIs in parallel with vaccine rollout. With less than 6% of the country previously infected, naturally acquired immunity was minimal before the surge [7]. In addition, vaccine-induced immunity in the population was likely much lower than that expected from the coverage due to imperfect vaccine efficacy [8]. There is particular uncertainty regarding the efficacy of the Sinopharm vaccine given that the clinical trial results have not been published in a peer-reviewed journal. The rise in infections soon after the relaxation of NPIs indicates that the population immunity had not reached the herd immunity threshold, which likely exceeds 70% [9]. Other challenges to the attainment of herd immunity are the reduced vaccine efficacy against some variants of SARS-CoV-2 with greater transmissibility [10], necessitating even higher coverage to avert future outbreaks. The crisis may be further exacerbated by behavioual changes from vaccinated people who believe they are fully protected and become less cautious about SARS-CoV-2 transmission.

Our analysis shows that easing NPIs prematurely risks a COVID-19 resurgence, which would hinder progress towards global pandemic control where significant disparities in vaccine distribution remain [11]. Given the variability in pandemic burden across the world, policy decisions on NPIs should be based on the local COVID-19 situation in each individual country, taking into consideration population-level immunity, vaccine efficacy and the relative prevalence and transmissibility of circulating variants.

## Additional material


Online Supplementary Document
