# Supplementary material for: Importance of non-pharmaceutical interventions in the COVID-19 vaccination era: A case study of the Seychelles
Source: J Glob Health. 2021 Sep 18;11:03104. doi: 10.7189/jogh.11.03104 (PMC8501397; doi:10.7189/jogh.11.03104)
Supplement: Online Supplementary Document [file jogh-11-03104-s001.pdf]

Appendix S1

Importance of non-pharmaceutical interventions in the COVID-19 vaccination era: a case study of Seychelles

Thomas N. Vilches,[1] Pratha Sah,[2] Elaheh Abdollahi,[2] Seyed M. Moghadas,[2] Alison P. Galvani[1]

[1] Center for Infectious Disease Modeling and Analysis (CIDMA), Yale School of Public Health, New Haven, Connecticut 06520, USA

[2]Agent-Based Modelling Laboratory, York University, Toronto, Ontario, M3J 1P3 Canada

This Appendix provides details of the methodology for back-calculation of incidence and the simulation model.

We used a dynamic agent-based transmission model calibrated to reported cases in the Seychelles to examine the cause behind the recent surge in COVID-19 cases, and the role of vaccination and non-pharmaceutical interventions (NPIs) in the country. Timelines for non-pharmaceutical interventions adopted in the Seychelles are shown in Figure S1.

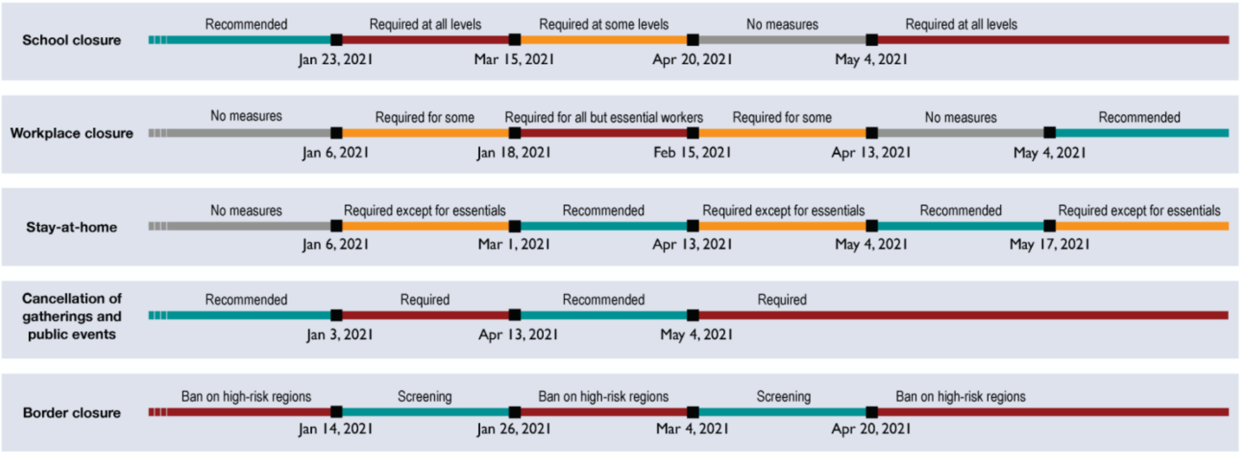

Figure S1. Timelines of non-pharmaceutical interventions implemented during the 2021 COVID-19 outbreak in the Seychelles. Vaccination against COVID-19 started on January 13, 2021.

Back-calculation of incidence

We used a Bayesian non-parametric approach to back-calculate the times series of infections based on the daily reported cases of COVID-19 in the Seychelles from October 21, 2020 to May 24, 2021.[1] Letting I_i represent the number of infections in the ith time interval, the reported cases on day i, D_i, can satisfy the convolution equation: [2,3]

$$D_i = \sum_{j=1}^{i} I_j p_{i-j}$$

where p_{i-j} is the probability that the time between infection and identification is $i-j$. Considering the incubation period as a proxy for time from infection to identification, p_{i-j} can be

directly calculated from the incubation period distribution. Here we make a simplifying assumption that the distribution of the incubation period does not change over time.

We employed a Bayesian approach to estimate posterior densities of the expected incidence of infections in time i in a random walk simulation process. The observed number of infections at time i (the calendar day i) was modelled by a nonhomogeneous Poisson process: $I_i \sim \mathrm{Poisson}(\lambda_i)$ where prior distributions for the λ_i were uninformative given by $\lambda_1 = \mathrm{Uniform}(0, 10^3)$, and $\lambda_i \sim \mathrm{Normal}(\lambda_{i-1}, \sigma^2)$, for $i \geq 1$ in the truncated distribution for $\lambda_i > 0$. The standard deviation, σ, was set to 10 for our analyses to maintain some variation. Back calculation was implemented in a Bayesian Markov Chain Monte Carlo (MCMC) setting using Nimble, with the R statistical environment acting as the front end. MCMC simulations were run in 5 independent chains, each consisting of 25,000 iterations, with a burn-in period of 10,000 iterations and a thinning factor of 10. To assess convergence, we inspected the trace plots and applied the Gelman-Rubin convergence test by computing the potential scale reduction factors (PSRF) of posterior densities. Figure S1 shows the simulation results for the cumulative incidence of infection with the 95% credible interval.

Agent-based modelling

We fitted an agent-based model of COVID-19 transmission to incidence derived from back-calculation from October 21, 2021, to January 6, 2021. In this period, there were no school or workplace closures, and non-pharmaceutical interventions for stay-at-home, and cancelation of gatherings remained mainly at the “Recommended” level. Border restrictions included a ban on high-risk regions for International travelers (Figure S1). We determined disease transmissibility by fitting the model to incidence data during this period while accounting for mask-wearing and recommended measures to reduce contact patterns to 80% of the pre-pandemic behaviour. The model simulated scenarios of COVID-19 incidence without vaccination, and when vaccination was implemented on January 13, 2021.

Model structure

We adopted our previous agent-based model [4] with the natural history of COVID-19, including epidemiological classes for susceptible; latently infected (not yet infectious); asymptomatic (and infectious); pre-symptomatic (and infectious); symptomatic (and infectious) with either mild or severe illness; recovered; and dead. The model population was stratified into six age groups of 0-4, 5-19, 20-49, 50-64, 65-79, and 80+ years based on demographics of the Seychelles.[5] Daily contacts between individuals were sampled from a negative-binomial distribution parameterized using an empirically-determined age-specific contact network.[6,7]

Disease dynamics

We parameterized the transmissibility of asymptomatic, mild symptomatic, and severe symptomatic stages to be 26%, 44%, and 89% relative to the pre-symptomatic stage.[8–10] The incubation period was sampled from a log-Normal distribution [LogN(shape: 1.434, scale: 0.661)] with a mean of 5.2 days.[11] For those who developed symptomatic disease, the pre-symptomatic stage was sampled from a Gamma distribution [Gamma(shape: 1.058, scale: 2.174)] with a mean of 2.3 days.[9,12] The infectious period following the onset of symptoms was

sampled from a Gamma distribution [Gamma(shape: 2.768, scale: 1.1563)] with a mean of 3.2 days.[9,13] Those who did not develop symptomatic disease remained asymptomatic until recovery, with an infectious period that was also sampled from a Gamma distribution [Gamma(shape: 5, scale: 1)] with a mean of 5 days.[13,14] We assumed that recovery from infection conferred immunity against reinfection for at least one year. We also assumed that all symptomatic cases who were not hospitalized self-isolated within 24 hours of symptom onset, and reduced their number of daily contacts by an average of 75%.

Vaccination

We implemented a two-dose vaccination campaign from January 13, 2021, and used the weekly average of vaccine doses distributed since the start of vaccination to simulate the vaccination scenario.[1] Two different vaccines used in the Seychelles included Sinopharm (with a 21-day between doses interval) and AstraZeneca (with a 28-day between doses interval). Among fully vaccinated individuals by May 8, 2021, 57% had received Sinopharm vaccines and 43% were vaccinated with AstraZeneca shots.[15] Since data on efficacy of Sinopharm vaccines are limited, we used efficacy data of AstraZeneca vaccines as the best-case scenario, and considered vaccine efficacy of 76.0%, (95% CI: 59.0% - 86.0%) and 81.5%, (95% CI: 67.9% - 89.4%) against symptomatic disease 14 days after first and second doses, respectively; 100% against severe disease 14 days after the first dose; and 51.9%, (95% CI: 42.0% - 60.1%) and 77.3% (95% CI: 65.4% - 85.0%) against infection 14 days after the first and second doses, respectively.[16–21]

Model implementation

We simulated the model with a population of 98000 individuals, assuming no pre-existing immunity. Results were generated by averaging 500 independent Monte-Carlo realizations in each scenario, and credible intervals (CrI) were generated using the bias-corrected and accelerated bootstrap method (with 500 replications). The model was implemented in Julia, which is an open-source, high-performance, dynamic programming language. The simulation codes are available at: https://github.com/thomasvilches/Seychelles

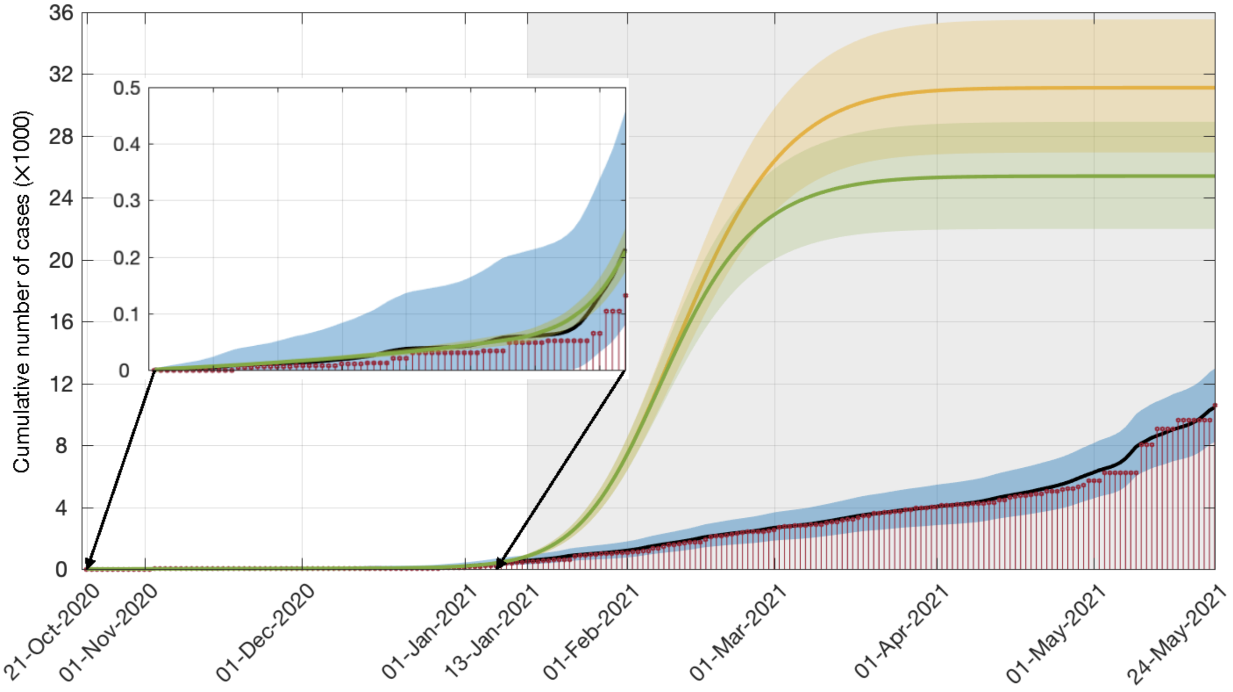

Figure S2. The cumulative mean of posterior distributions for infections (black) back-calculated by fitting the convolution equation to daily reported cases (red). The 95% credible interval is represented by a blue shaded area. Simulated outbreaks using the agent-based model (fitted to cumulative infections till January 6, 2021) are shown for the cumulative number of infections without vaccination (orange curve), and with a two-dose vaccine series (green curve). Shaded orange and green areas represent the 95% credible intervals of simulated outbreaks. Grey area represents the vaccination era.

References

1. covid-19-data. Github https://github.com/owid/covid-19-data (accessed June 4, 2021).
2. Sun X, Nishiura H, Xiao Y. Modeling methods for estimating HIV incidence: a mathematical review. *Theor Biol Med Model* 2020; 17: 1.
3. Fraser C. Estimating individual and household reproduction numbers in an emerging epidemic. *PLoS One* 2007; 2: e758.
4. Sah P, Vilches TN, Moghadas SM, *et al.* Accelerated vaccine rollout is imperative to mitigate highly transmissible COVID-19 variants. *EClinicalMedicine* 2021; 35: 100865.
5. United Nations Statistics Division. Demographic and Social Statistics. https://unstats.un.org/unsd/demographic-social/products/dyb/index.cshtml (accessed June 5, 2021).
6. Jarvis CI, Van Zandvoort K, Gimma A, *et al.* Quantifying the impact of physical distance measures on the transmission of COVID-19 in the UK. *BMC Med* 2020; 18: 124.
7. Mossong J, Hens N, Jit M, *et al.* Social contacts and mixing patterns relevant to the spread of infectious diseases. *PLoS Med* 2008; 5: e74.
8. Sayampanathan AA, Heng CS, Pin PH, Pang J, Leong TY, Lee VJ. Infectivity of asymptomatic versus symptomatic COVID-19. *Lancet* 2021; 397: 93–4.

9. Moghadas SM, Fitzpatrick MC, Sah P, *et al.* The implications of silent transmission for the control of COVID-19 outbreaks. *Proc Natl Acad Sci U S A* 2020; 117: 17513–5.
10. Ferretti L, Wymant C, Kendall M, *et al.* Quantifying SARS-CoV-2 transmission suggests epidemic control with digital contact tracing. *Science* 2020; 368. DOI:10.1126/science.abb6936.
11. Li Q, Guan X, Wu P, *et al.* Early Transmission Dynamics in Wuhan, China, of Novel Coronavirus–Infected Pneumonia. *N Engl J Med* 2020; 382: 1199–207.
12. He X, Lau EHY, Wu P, *et al.* Temporal dynamics in viral shedding and transmissibility of COVID-19. *Nat Med* 2020; 26: 672–5.
13. Li R, Pei S, Chen B, *et al.* Substantial undocumented infection facilitates the rapid dissemination of novel coronavirus (SARS-CoV-2). *Science* 2020; 368: 489–93.
14. Gatto M, Bertuzzo E, Mari L, *et al.* Spread and dynamics of the COVID-19 epidemic in Italy: Effects of emergency containment measures. *Proc Natl Acad Sci U S A* 2020; 117: 10484–91.
15. Hollingsworth J. The Seychelles is 60% vaccinated, but still infections are rising. That's not as bad as it sounds. *CNN* 2021; published online May 15. https://www.cnn.com/2021/05/14/africa/seychelles-covid-vaccination-infection-intl-hnk-dst/index.html (accessed June 4, 2021).
16. Emary KRW, Golubchik T, Aley PK, *et al.* Efficacy of ChAdOx1 nCoV-19 (AZD1222) vaccine against SARS-CoV-2 variant of concern 202012/01 (B.1.1.7): an exploratory analysis of a randomised controlled trial. *Lancet* 2021; 397: 1351–62.
17. COVID-19 Vaccine AstraZeneca confirms 100% protection against severe disease, hospitalisation and death in the primary analysis of Phase III trials. 2021; published online Feb 3. https://www.astrazeneca.com/media-centre/press-releases/2021/covid-19-vaccine-astrazeneca-confirms-protection-against-severe-disease-hospitalisation-and-death-in-the-primary-analysis-of-phase-iii-trials.html (accessed June 5, 2021).
18. Vasileiou E, Simpson CR, Shi T, *et al.* Interim findings from first-dose mass COVID-19 vaccination roll-out and COVID-19 hospital admissions in Scotland: a national prospective cohort study. *Lancet* 2021; 397: 1646–57.
19. Knoll MD, Wonodi C. Oxford-AstraZeneca COVID-19 vaccine efficacy. Lancet. 2021; 397: 72–4.
20. Voysey M, Clemens SAC, Madhi SA, *et al.* Safety and efficacy of the ChAdOx1 nCoV-19 vaccine (AZD1222) against SARS-CoV-2: an interim analysis of four randomised controlled trials in Brazil, South Africa, and the UK. *Lancet* 2021; 397: 99–111.
21. Vizient, Inc. COVID-19 vaccine candidates. 2021 https://www.vizientinc.com/-/media/documents/sitecorepublishingdocuments/public/covid19_sidebyside_vaccinecompare.pdf.
